# Within- and between-person factor structure of the Oldenburg Burnout Inventory: Analysis of a diary study using multilevel confirmatory factor analysis

**DOI:** 10.1371/journal.pone.0251257

**Published:** 2021-05-14

**Authors:** Ewa Gruszczynska, Beata A. Basinska, Wilmar B. Schaufeli

**Affiliations:** 1 Faculty of Psychology, SWPS University of Social Sciences and Humanities, Warsaw, Poland; 2 Faculty of Management and Economics, Gdansk University of Technology, Gdansk, Poland; 3 Faculty of Psychology, Netherlands and Faculty of Psychology, Utrecht University, KU Leuven, Belgium; University of Copenhagen, DENMARK

## Abstract

The study examined the factor structure of burnout, as measured with the Oldenburg Burnout Inventory. The participants were 235 employees of a public administration agency who assessed their burnout online for 10 consecutive working days. Two models were tested with multilevel confirmatory factor analysis, assuming the same one or two-factor structure at the within- and between-person levels. Both models showed a reasonable fit to the data, but due to a strong correlation between exhaustion and disengagement and low within-person reliability for disengagement, a unidimensional model seems more valid. A cross-level invariance was not confirmed for either of the structures, showing that factor loadings for the same items differ significantly between the levels. This suggests that burnout is not the same latent variable at each level; rather, there are factors other than daily burnout that influence person-level scores and ignoring these across-level discrepancies may lead to biased conclusions.

## Introduction

Job burnout, which refers to job stress that has not been properly managed, is a wide-spread phenomenon associated with the contemporary work. Recently the World Health Organization [1] included burnout in the latest version of the International Classification of Disease (ICD-11), illustrating the global importance of this “occupational phenomenon”. During working life, two-thirds of full-time employees suffer from job burnout [2], citing low job satisfaction, disappointing job performance and poor relationships with co-workers and supervisors as reasons for job burnout. Experiencing burnout is tightly related to work; however, its consequences may spill over to other psychological and social aspects of life. Although burnout seems to be a more serious problem among human service professionals than other professionals [3–6], it can be observed in any profession. Thus, a more precise identification of the burnout phenomenon among employees, from early signs to clinical symptoms, is important for the welfare of society as a whole.

Until recently, the vast majority of findings on burnout were obtained from cross-sectional studies, resulting in attention being paid mainly to *between-person differences* that reflect stable interpersonal characteristics [7–9]. However, an increasing number of aspects of well-being have been conceptualised as dynamic states that fluctuate from one day to another for the same person, providing unique insight into *within-person variability*. Study designs based on frequently repeated measurements in the same person with a relatively short time interval, such as diary studies [10], provide an opportunity to decompose observed variability into a stable and changing component for a given person [11]. This means that diary studies reflect the complexity of human behaviour in everyday life, where reactions are driven by trait-like, time-invariant characteristics and contextual, time-variant factors. Additionally, because both of these levels are derived from daily reports, the impact of retrospective recall bias is reduced [11–13].

In this study, we focus on a suchlike two-level structure of job burnout, using an intensive longitudinal design. Specifically, we will examine the within- and between-person factor structure of job burnout using the Oldenburg Burnout Inventory (OLBI) in a 10-day diary study. To our best our knowledge, our study unique because it is the first to test multilevel measurement invariance of burnout.

### Structure of burnout syndrome

#### Maslach and Jackson’s theory and measurement

The most popular conceptualization of job burnout is in line with Maslach and Jackson’s [14] definition, which considered burnout to be a chronic state of exhaustion, depersonalisation, or cynicism and a lack of personal accomplishment. Initially job burnout syndrome was identified in client-service professionals but later it was reformulated so that it could also be observed in other workers who do not personally engage in customer relations to perform their job [15]. Thus, the syndrome of burnout was described as comprising three universal dimensions: exhaustion, cynicism and reduced professional efficacy.

A review and meta-analysis of 45 studies on exploratory and confirmatory factor analyses of the structure of burnout underlying the Maslach Burnout Inventory (MBI) [16] revealed that most studies only examined the three-factor model proposed by Maslach and Jackson [14]. Surprisingly, only one study [17] tested a sequence of models that included from one to five factors. Overall, the reported studies on the confirmatory factor analysis of the MBI provided most consistent support for the correlated three-factor model of burnout, but two-factor and five-factor structures were also found. Specifically, Kalliath et al. [18] confirmed a two-factor model that consisted of emotional exhaustion and depersonalisation as distinct factors, but excluded all of the items that originally formed the personal accomplishment subscale. De Beer and Bianchi [19] demonstrated that a model consisting of a *combined* emotional exhaustion and depersonalization factor, and a separate personal accomplishment factor fitted the data best. In contrast, Densten [17] verified a five-factor model in which both the emotional exhaustion and personal accomplishment subscales formed two distinct factors. Moreover, the correlation between emotional exhaustion and depersonalisation was consistently positive and moderate, compared to the inconsistent relationships with personal accomplishment [16].

Exhaustion has been characterized by a loss of energy, resources depletion, cognitive wearing out, and fatigue [21,22], which is a broader understanding compared to the earlier proposal by Maslach and Jackson [14]. In line with the processual nature of “burning out,” exhaustion is assumed to develop first, as a response to excessive job demands and work overload [22]. In this light, the common opinion that exhaustion is a critical component of burnout is unsurprising [20–22]. Another argument supporting this view was provided in a study by Schonfeld et al. [23] using exploratory structural equation modelling bifactor analysis. The results revealed that emotional exhaustion, depression and anxiety could be combined into one general factor labelled nonspecific psychological distress. The depersonalisation items loaded similarly on two factors, i.e. nonspecific psychological distress and depersonalisation, and personal accomplishment items were only weakly related to the general factor [23]. This suggests that exhaustion shares common ground with depersonalization, but not with personal accomplishment, which may be more important for the development of a clinical, symptomatic stage of burnout. In fact, depersonalization may follow exhaustion, as it consists of negative reactions to social relationships as well as aversive attitudes toward work [22]. Thus, a lack of personal accomplishment can be viewed as a *consequence* of both exhaustion and depersonalization, since the combination of being exhausted and distancing oneself from others leads to poor job performance, reduced productivity, and decreased job satisfaction [22]. This indicates why two-dimensional structure could be regarded as the most valid model for burnout as it separates the underlying symptoms from their effects [24–28].

In addition, there also is a theoretical argument for including only exhaustion and depersonalization into the burnout syndrome. Schaufeli and Taris [29] in reference to an idea of psychological fatigue as the intolerance of any effort, assumed that the *inability* and the *unwillingness* to spent effort at work constitute two facets of burnout. It means that burnout has an energetic (exhaustion) and motivational component (distancing from work). In light of these findings, the two-factor structure is promising in understanding work under stress and introducing appropriate intervention.

The job burnout structure has not yet been examined before using a multilevel confirmatory factor analysis. The multilevel approach in job burnout studies usually regards differences between levels of hierarchical management structures [e.g., 30,31] and relationships between professionals and their clients or patients [e.g., 32,33], rather than multiple measurements nested within a person.

#### Job demands-resources theory and measurement

According to the Job Demands-Resources (JD-R) model, burnout is a two-dimensional syndrome comprising exhaustion and disengagement, which is defined as a prolonged negative state of well-being caused by a demanding work environment [23,25]. Exhaustion is described as “a consequence of intensive physical, affective and cognitive strain, that is, as a long-term consequence of prolonged exposure to certain job demands” [27, p. 201]. Disengagement is characterised by “distancing oneself from one’s work in general, work object, and work content” [27, p. 210–211]. In this understanding, exhaustion affects multiple aspects of functioning and disengagement is a broad negative attitude toward work, closely related to cynicism [23,25]. In contrast, depersonalization is a narrower term, describing only one form of disengagement with a focus on human relations at work, particularly with clients [25]. As mentioned by Schaufeli and Taris [29], exhaustion reflects the energy components of job burnout, and disengagement refers to identification with one’s work. The former is important to the health impairment mechanism, and the latter to the process of motivational deterioration [34]. The findings of a meta-analysis by Lesener et al. [35] suggest that the JD-R model is an excellent theoretical basis to assess employee well-being across a broad range of organisations and jobs.

The OLBI is an instrument to measure burnout syndrome in the framework of the JD-R theory [25]. The main advantage of the OLBI over the MBI lies in its origin. Namely, the MBI was constructed inductively (i.e., data-driven process), whereas the OLBI was constructed deductively (i.e., theory-driven process). Also, OLBI is available in open access form. Some of the very first studies using the OLBI were conducted in Greek and German employees from different occupational groups [25,26,36]. After twenty years, free versions of the OLBI are available in many languages and from most continents, including versions from Europe, North and South America [37–39], Asia [40,41], Africa [42] and Australia [43,44].

A proposed two-factor model of the OLBI, consisting of exhaustion and disengagement, was empirically supported in samples such as working adults and fire department employees in the United States [37], German employees and German and Greek students [45] and workers at a cement factory in the North West province of South Africa [42].

Sinval et al. [39] reviewed the structure of the OLBI based on 14 published papers presenting results of confirmatory factor analysis. Most of them indicated that a two-factor structure has a good fit to the data and performed better than a one-factor structure. For example, a two-factor model of the Tamil version of the OLBI had an acceptable fit in two independent samples (police constables and higher secondary teachers) compared to a unidimensional model [41]. However, the correlation between the two factors was substantial, equalling .67 and .65, respectively. Similar findings were demonstrated in German nurses [45] and a multi-occupational sample in Sweden [46].

Thus, despite the many advantages of the OLBI, the moderate to high correlations between exhaustion and disengagement may suggest a lack of discriminant validity [47]. Most likely, this is at least partially caused by the positive and negative wording of the items [48]. Originally, in each subscale, half of the items described negative states and the other half positive ones. As a result, the obtained factor structures coincided more with the way the statements were formulated than with the theoretically assumed factors. Research findings support an exhaustion–disengagement model, with only the negatively framed items [49], or a four-factor model in which exhaustion and disengagement are divided into positively and negatively worded factors [25,40,49]. Seen from this perspective, positively worded items can be regarded as referring to work engagement and as such may represent either the opposite, positive pole of burnout or the concept independent from burnout [50,51]. Thus, a version including only negatively framed items, as proposed by some researchers [47,49], is most valid to measure burnout, particularly in intensive longitudinal designs, such as diary studies, in which short instruments are necessary to reduce the burden on participants [52].

### Job burnout as trait versus state

The traditional view of burnout as a chronic and relatively stable syndrome has been challenged by findings from diary studies. Although a growing interest in daily symptoms of burnout has been observed recently [53–55], the measurement of momentary burnout is still in its infancy [56]. The within-person approach suggests that burnout symptoms may vary within the same employee from one day to another; thus, these symptoms could be described as a dynamic state, changing over time [57–59]. Within-person studies have primarily focused on psychological detachment as a predictor of strain or ill-being. For this reason, job burnout is often reduced to exhaustion [60,61]. A review of within-person studied showed that 33% to 65% of the variance in job burnout could be assigned to within-person factors and 35% to 77% to between-person components [52]. Thus, alongside well-recognised interpersonal differences in burnout, short-term within-person variability is also substantial and worthy of more systematic investigation. The main limitation of these within-person studies is that they mostly used the MBI to measure burnout. We found only five daily diary studies, in which symptoms of burnout were assessed with the OLBI [62–66], though these were mainly limited to measurement of exhaustion. Only two assessed both components of burnout, but the OLBI was shortened to 12 [63] and 6 items [65], without further specifying them.

Consistency of factor structure at both the between- and within-person levels simultaneously has not yet been tested for either MBI or OLBI. The within-person structure is simply assumed to be the same as that of more traditional between-person designs that focus on self-reports of how a person *typically* feels. The burnout patterns observed between individuals, however, do not necessarily translate into intra-individual processes. Such an approach can lead to erroneous conclusions as it is based on an unverified conviction that burnout components at within- and between-person levels are measurably identical. Therefore, the current study examines the factorial validity of the OLBI at both the between-person (trait-like) and within-person (state-like) levels to fill this gap. This is of special importance in light of the increasing popularity of daily assessments of burnout, also as predictors or outcomes of other processes.

## Materials and methods

### Participants

Of the 238 employees of a public administration agency recruited for the present study, 235 (73% women) participated in all the measurement points. They provided complete data as missing data in daily diary studies usually occurs in the form of dropout or omission of the whole survey on a given day [10]. Participants were engaged in service provision to the general public (87% on a permanent basis). The participants were between 21 and 68 years old (mean (*M*) = 38 years, standard deviation (*SD*) = 9.7) and most of them had a higher education degree (81%). They mostly declared being married or cohabiting with a partner (77%). Participants reported up to 43 years of job tenure (*M* = 15 years, *SD* = 10.4) and up to 27 years of work in their current position (*M* = 7.5 years, *SD* = 6.3). Of the employees who participated in the study, 15% held a managerial position.

### Procedure

The study was conducted in accordance with applicable ethical rules (the Declaration of Helsinki) and was approved by the Committee on Research Ethics of SWPS University of Social Sciences and Humanities, Warsaw, Poland; decision no. 41/2016. A professional surveying agency recruited the participants. The trained research assistants contacted the heads of public administration units and presented the study’s aims and protocol by phone or, if requested, by email. Potential participants were then contacted individually by email or phone, according to their preferences. Four inclusion criteria were used: engaged in service provision to the public, permanent contract, full-time employment and job tenure over 6 months. After obtaining preliminary consent, each participant received a letter of intent and a written informed consent form, which included remuneration details. The written informed consent was obtained from all the participants. The diary measurement was conducted online over a period of 10 consecutive working days (from Monday to Friday). The measurement took place each day after working hours were officially over. After completing the entire 10-day survey, participants received a remuneration of 30 euro and a letter of gratitude. The current study is a part of a larger longitudinal project on burnout and its correlates.

### Measure

Daily job burnout symptoms were as assessed using the 8-item OLBI [27]. Originally, the OLBI consisted of 16 items that included both positively and negatively worded items and focused on the measurement of burnout and engagement (as negative and positive aspects of well-being, respectively) [27]. Because we wanted to evaluate “pure” burnout, not contaminated by positive engagement items that should be reversed, we decided to use only the negatively worded items.

The items were reworded according to the specifics of the daily study of exhaustion (e.g. “Today, after my work, I felt worn out and weary”) and disengagement (e.g. “Today, I thought less at work and did my job almost mechanically”). Each item was rated on a five-point scale that ranged from 1 (*completely disagree*) to 5 (*completely agree*). Higher scores indicate higher daily burnout symptoms.

### Data analysis

The analysis was conducted in two steps. We started with multilevel confirmatory factor analysis (MCFA) [67] to test a two-factor model of burnout for diary data. We assumed that this two-factor model could be corroborated at each level of analysis; that is, at the between-person level, which describes variance related to more stable interpersonal differences (trait-like), and at the within-person level, which describes variance related to daily changes from what is typical for a given person (state-like). In terminology provided by Stapleton et al. [68], this is a configural cluster construct model in which clusters (here, persons) aggregate constructs at the daily level (e.g. average exhaustion for a given person across all days of the study). For such models, cross-level invariance constraints are required for the interpretation of the results at both levels as components of the same latent variables.

Thus, we tested invariance across levels with three increasingly restrictive models [69]. The first model did not include any additional constraints. The next model, with cross-level invariance, assumed the (unstandardized) factor loadings to be equal across both levels and freely estimated the factor variances at the between-person level. We additionally differentiated between weak and strong metric invariance according to Tay, Woo, and Vermunt’s [70] definition, in which a weak metric invariance is obtained when relative ordering of factor loadings holds between levels, and a strong metric invariance is obtained when magnitude of factor loadings holds between levels. In the third model, with strong factorial invariance, the residual variances at the between-person level were fixed at zero.

To assess goodness of fit, we used several indicators. We reported the chi-square (χ^2^) test value, which should be insignificant to indicate good fit. This parameter, however, is related to sample size and is therefore currently not considered decisive. For both the Comparative Fit Index (CFI) and the Tucker-Lewis index (TLI), values above .95 indicate good fit, whereas values above .90 but below .95 indicate adequate fit. The Root Mean Square Error of Approximation (RMSEA) and the Standardized Root Mean Square Residual (SRMR) with values up to .08 are generally considered to indicate a good fit [71,72]. However, among the indices mentioned above, only SRMR assesses fit at each level separately [67]. Therefore, we also reported Akaike Information Criterion (AIC), Bayesian Information Criterion (BIC) and the Sample size-Adjusted BIC (SABIC) as comparative fit indices to choose between competitive models with different numbers of factors. For these three indices, lower values indicate better fit.

For each solution, we also obtained omega (ω) to asses multilevel reliability [73]. This index has values ranging from 0 to 1, where values closer to 1 in indicate better internal consistency [for a review, see 74]. All computations for MCFA, omega included, were performed using Mplus version 8.2 [75].

## Results

### Descriptive statistics and intra-class correlation

Descriptive statistics for all OLBI items and indicators are provided in Table 1. The intra-class correlation (ICC) coefficients suggest that a significant proportion of variance in each variable is due to differences between persons. Specifically, with 40% and 41%, respectively, item 1D (*Today*, *more and more often I talked about my work in a negative way*) and item 6D (*Sometimes I felt sickened by my work tasks today*) have the highest proportions of between-person variance. Overall, within-person variance in daily burnout ranged from 59% to 70%.

**Table 1:** Univariate higher-order moment descriptive statistics and intra-class correlations for the OLBI items.

Items	Mean	SD	Skewness	Kurtosis	ICC
Item 1D. Today more and more often I talked about my work in a negative way.	1.98	1.05	.93	.16	.40
Item 2E. Today after work, I tend to need more time than in the past in order to relax and feel better.	2.40	1.17	.53	-.65	.32
Item 3D. Today, I thought less at work and did my job almost mechanically.	2.33	1.23	.42	-.68	.30
Item 4E. Today during my work, I felt emotionally drained.	2.45	1.16	.36	-.84	.33
Item 5D. Today over time, I could become disconnected from this type of work.	2.41	1.09	.36	-.73	.28
Item 6D. Sometimes I felt sickened by my today work tasks.	1.69	0.96	1.31	1.01	.41
Item 7E. Today after my work, I felt worn out and weary.	2.51	1.23	.37	-.92	.33
Item 8E. Today I felt tired before I arrived at work	2.04	1.10	.89	-.07	.36

Note. E = exhaustion; D = disengagement; SD = standard deviation; ICC = intra-class correlation.

### Structure of daily burnout symptoms: Multilevel confirmatory factor analyses

Results of MCFA are presented at Table 2. The goodness of fit indices indicate an acceptable fit for a multilevel two-factor structure of burnout. However, the very high correlations between disengagement and exhaustion at both the within-person (.94, *p* < .001) and between-person (.90, *p* < .001) levels may indicate a lack of discriminant validity between the factors, which requires an examination of the possible unidimensionality of burnout. Thus, we also tested a multilevel one-factor structure and found that it has very similar factor loadings. Also, the goodness of fit indices are comparable to the ones obtained for the two-factor model, with only slightly higher values for AIC, BIC and SABIC. Interestingly, the highest loadings for the two-factor and one-factor solutions were for the same two exhaustion items: item 4E (*Today during my work*, *I felt emotionally drained*) and item 7E (*Today after my work*, *I felt worn out and weary)*, suggesting that these are the strongest indicators of burnout at both levels.

**Table 2:** Summary of the results of multilevel confirmatory factor analysis: Two-factor model and one-factor model (unstandardized factor loadings).

Items	Two-factor model		One-factor model	
	Within-person	Between-person	Within-person	Between-person
	Disengagement			
Item 1D. Devaluation of work	.44	.55	.43	.53
Item 3D. Mechanical execution	.39	44	.37	.41
Item 5D. Inner relationship	.69	.55	.66	.52
Item 6D. Sick about work tasks	.40	.49	.39	.48
	Exhaustion			
Item 2E. Longer times for rest	.71	.58	.71	.57
Item 4E. Emotionally drained	.79	.63	.79	.63
Item 7E. Worn out	.78	.70	.78	.68
Item 8E. Tired before work	.42	.53	.42	.54
χ2 (df)	330.12* (38)		387.19* (40)	
CFI	.936		.926	
TLI	.905		.893	
AIC	55934		46012	
BIC	46177		46243	
SABIC	46043		46116	
RMSEA	.058		.061	
SRMR	.036	.058	.038	.057

*Note*. Omega values for two-factor model: Within-person .66, .81, and between-person .90, .95. for disengagement and exhaustion, respectively; for the one-factor model: Within-person .85, between-person .95. All the abbreviations explained in the data analysis section.

* *p* < .05.

Additionally, a two-factor model with correlations between factors fixed to zero fitted significantly worse to the data (χ2 = 1714.13, df = 40, *p* < .001, CFI = .631, RMSEA = .133, SMRS within = .26 and between = .42). Therefore, given only small differences between the correlated two-factor model and the one-factor model and the more parsimonious structure of the latter, burnout seems to be a unidimensional construct in our sample. This model is also supported by higher internal consistency at the within-person (ω = .85) and between-person (ω = .95) levels, whereas reliability for disengagement at Level 1 (ω = .66) in the two-factor model was below satisfactory.

### Cross-level measurement invariance

In the next step, measurement invariance was tested for both models. The parameters for nested model comparisons are provided in Table 3. Assuming a model without any additional constraints fitted reasonably well to the data, the next model with factor loading set equal across levels fitted significantly worse for both the two-factor model (Sattora–Bentler scaled Δ χ2 = 50.72, df = 8, *p* < .001) and the one-factor model (Sattora–Bentler scaled Δ χ2 = 48.27, df = 8, *p* < .001). This means that factor loading values at the within- and between-person levels, presented in Table 3, differ significantly, irrespective of whether the model has one or two factors.

**Table 3:** Multilevel measurement invariance: A comparison of nested models for the two- and one-factor solutions.

Models	df	χ2	RMSEA	SRMR		CFI	BIC
				w	b		
*Two-factor model*							
1. No constraints	38	330.12	.058	.036	.058	.936	46177
2. Cross-level invariance	46	381.19	.056	.039	.132	.926	46183
3. Strong factorial invariance	54	1606.43	.110	.098	.235	.858	47387
*One-factor model*							
1. No constraints	40	387.19	.061	.038	.057	.926	46057
2. Cross-level invariance	48	435.99	.059	.040	.138	.914	46248
3. Strong factorial invariance	56	2165.03	.126	.117	.287	.535	47872

*Note*. w = within, b = between. Other abbreviations explained in the in the data analysis section. For each model χ2 significant at *p* < .001.

Furthermore, based on all of the indices, the next model in the hierarchy with residual variance fixed at 0 at the between-person level can be regarded as fitting the data much worse than the first two models. Collectively, the results show a lack of multilevel measurement invariance. This suggests that: 1) burnout is not represented by the same latent variable at each level, and 2) there are factors other than daily burnout influencing the person-level scores on the items.

Even when following the suggestion by Tay, Woo, and Vermunt [70] to use weaker forms of cross-level invariance not based on the exact values, but on the same rank order of factor loadings across levels, the factor loadings in our study violated this rule for three items (1D, 5D, 4E) in the one-factor solution and for one item (4E) in the two-factor solution. Thus, this again indicated a lack of cross-level measurement invariance.

## Discussion

The goal of our study was to examine the structure of burnout as assessed by the OLBI, using the data of a 10-day diary study. We found that both factor models analysed, one using a theoretically driven two-factor structure (exhaustion and disengagement) and the other using a single-factor structure, fitted reasonably well to the data. However, a strong correlation between exhaustion and disengagement at the within- and between-person levels suggested that a one-factor model was more appropriate. Additionally, burnout seems represented more strongly by items tapping exhaustion than disengagement.

Therefore, our results indicate that job burnout can be viewed as a general single-factor construct in a multilevel approach based on daily assessments. This contradicts the view of burnout as a multidimensional phenomenon [16,39], but it agrees with an idea of burnout as a syndrome, that consists of interrelated symptoms referring to one underlying state or condition [34,76]. This assumption is also reflected in the studies operationalizing burnout in terms of interindividual differences with a single aggregated indicator [63], as well as in the clinical practice [19].

Moreover, exhaustion items seem to comprise an accurate and coherent indicator of daily symptoms of burnout. This is consistent with the established concept of exhaustion as a critical component of job burnout [21,22] and as a central pathogenic health indicator within the JD-R theory [35,76]. Similarly, in the recent debate on job burnout, an international panel of experts agreed that “in a worker, occupational burnout or occupational physical and emotional exhaustion state is an exhaustion due to prolonged exposure to work-related problems" [77, p. 11]. Still, the exact nature of exhaustion remains unclear, particularly with regard to the question of it being either a sufficient or necessary but insufficient condition of burnout [78].

Declining motivation to continue spending effort can be a protective strategy in case of acute psychological fatigue, facilitating recovery processes, especially on a daily basis [79]. In this light, disengagement may be secondary and reactive to daily exhaustion [52]. However, long-term disengagement is likely to become dysfunctional since it is associated with permanently impaired motivation, which, in its turn, further promotes exhaustion [80]. Taken together, this shows that micro- and macro- mechanism may diverge as they capture burnout processes in different time frames. Similarly, in numerous empirical studies, job burnout has been operationalized in terms of an energetic component as a depletion of vital resources [7,53,61]. Thus, in reference to the daily data collected in our sample, job burnout may be seen as general in nature, with exhaustion as its core aspect.

Additionally, we observed that two disengagement items, namely 3D (*Today*, *I thought less at work and did my job almost mechanically*) and 6D (*Sometimes I felt sickened by my today work tasks*), had particularly low factor loadings. A relevant comparative empirical material is lacking since no factor analysis was reported in the available studies on daily burnout [62–66]. Nonetheless, the results from classical confirmatory factor analysis [37,39,49] show that while the 3D item loadings indicate general satisfactory values (e.g., .64–.82), the range for the 6D item is less conclusive (e.g., .49–.76). Still, in these studies, not only was ‘trait-like’ burnout measured, but the length of the OLBI varied; thus, future studies should investigate this issue in greater detail. Special attention should be paid to whether low factor loadings of these items in diary studies on burnout will repeat regularly across different samples. If this were the case, it would require a deeper revision of the OLBI as a valid operationalization of ‘state-like’ burnout.

No cross-level measurement invariance was confirmed for either the one- or two-factor model. This may lead to problems in interpreting of the construct, since the same items at each level represent different saturation of the factor(s) [81]. As a consequence, it cannot be assumed that the same construct is measured at the within-person and between-person levels; that is, between-person differences in burnout are not simply a result of the aggregation of daily measures from each person. For instance, the item *Today during my work*, *I felt emotionally drained* is the strongest representation of burnout at the within-person level, but it is weaker at the between-person level. It is likely that individual differences in feeling emotionally drained at work can be caused by factors other than accumulation of daily experiences of this kind. When someone reports feelings as fluctuation over her or his typical level of being emotionally drained on one day, it does necessarily not lead to the conclusion that this person typically has the highest score on the trait-like measure of being emotionally drained. Thus, this item better captures these intrapersonal fluctuations than interpersonal differences, which are affected by other factors.

Therefore, it can be suggested that findings from studies focusing on differences between individuals, especially those using a cross-sectional design, should not be generalised for the understanding of within-person changes in job burnout [82]. For example, a meta-analysis of longitudinal studies [35] revealed a reciprocal effect between job characteristics and job burnout, but this relationship was stronger at the between-person than at the within-person level. Similar findings were confirmed in the recent meta-analysis of diary studies using 55 unique samples [83].

The question is whether these results may shed a new light on the nature of burnout, since it may have non-equivalent meanings for different people. Perhaps individuals construe experiences of burnout in different ways due to their stage of burnout development or other personality- or job-related characteristics. However, individuals with very high burnout levels are usually underrepresented in existing studies [84,85] so that most of our knowledge this comes from clinical samples. Nonetheless, this is still a relatively new topic that is generally overlooked in the literature dominated by a tacit assumption of construct isomorphism at each data level [70].

Our study has some obvious limitations that must be taken into account. First, sample specificity may influence the results, as a public administration agency provides a very stable working environment, which is highly predictable in terms of duties. Additionally, the major source of workplace stress among individuals working in such public agencies are demands caused by contact with citizens or interpersonal relationships within the office. Thus, the factor loadings and structure may not be transferable to other groups, especially since so far there has been no study that would make it possible to compare our results with those obtained in other workplace environments. Second, the number of measurement days could be insufficient to correctly represent within-person fluctuations. Finally, the results address burnout only as operationalised by OLBI, not by other measurement tools. Consequently, the highly homogenous population of public administration employees, small sample size, and study design make it unclear to what extent the results are transferable to other occupations, cultures, and burnout measures.

In conclusion, the results provide a positive indication for use of a single-factor model of the multilevel factor structure of burnout assessed using OLBI in a 10-day diary study. However, cross-level measurement invariance has not been confirmed for any of the models, suggesting discrepancies between within- and between-person interpretations of the construct. A person is an unexpected source of variability of the construct, which may lead to bias in comparisons of scores that ignore these differences.

These conclusions mentioned above have theoretical and practical implications. Theoretically, our conclusions emphasize the need for future research, taking into account the differences in the understanding of burnout and its development processes, depending on the level of analysis. Practically speaking our conclusions suggest that different items should be used to tap burnout *between* and *within* individuals. Also, an evaluation of daily burnout seems more likely to provide reliable data for monitoring the effectiveness of the organisation’s efforts in promoting a healthy work design and personal competences of their employees [86].
